# Procedural skills workshops for primary care physicians in Israel: a comprehensive analysis

**DOI:** 10.1186/s12909-024-05381-7

**Published:** 2024-04-10

**Authors:** Omer Rosenblum, Ilan Yehoshua, Limor Adler, Ori Liran

**Affiliations:** 1 Maccabi Healthcare Services, Tel Aviv, Israel; 2 Ben Gurion University, Beer Sheva, Israel; 3 Faculty of Medicine, Tel Aviv University, Tel Aviv, Israel

**Keywords:** Procedural skills, Manual skills, Primary care, Family physician, Education

## Abstract

**Background:**

Some of the most common complaints addressed by primary care physicians (PCPs) require manual procedures, such as lacerations repair, abscesses drainage, ingrown toenails removal, dry needling for myofascial pain syndrome, and Epley maneuver for treating benign paroxysmal positional vertigo (BPPV). The aim of this study was to describe the procedural skills workshops program for PCPs implemented in Maccabi Healthcare Services and to investigate how many PCPs have participated and used the skills since the program’s inception in 2017.

**Methods:**

In this observational study, we followed all participants in courses from 2017 to 2021. We extracted all procedures performed during these years by PCPs who learned the skill in MHS.

**Results:**

During the study period, 620 PCPs participated in workshops for dry needling, soft-tissue and joint injections, BPPV treatment, minor surgical procedures, and spirometry. Most procedures performed were dry needling (average annual number 3,537) and minor surgical procedures (average annual number 361). The average annual use per physician was highest for dry needling (annual average use per physician who used the learned skill was 50.9), followed by soft tissue and joint injections (16.8), minor surgical procedures (14.8), and BPPV treatment (7.5).

**Conclusion:**

procedural skills workshops may expand PCPs’ therapeutic arsenal, thus empowering PCPs and providing more comprehensive care for patients. Some manual skills, such as dry needling, soft tissue injections, and the Epley maneuver, were more likely to be used by participants than other skills, such as spirometry and soft tissue injections.

## Background

Primary care physicians (PCPs) are patients’ first point of contact for most symptoms and medical complaints. PCPs provide basic care for most patients’ health issues. Some of the most common complaints addressed by PCPs require manual procedures, such as lacerations, abscesses, ingrown toenails, myofascial pain syndrome, and benign paroxysmal positional vertigo (BPPV) [1–5]. The procedures include minor surgical procedures, joint injections, dry needling, the Epley maneuver, spirometry, etc. Many PCPs do not use these procedures regularly.

There are several advantages in performing manual procedures by PCPs: continuity of care, shorter waiting times, convenience to the patient, and financial benefit for the healthcare maintenance organization (HMO) since procedures conducted by the PCP are less expensive than those performed in a hospital [6]. Performing manual procedures by the PCP may also improve the patient-physician relationship, increase physicians’ work satisfaction, and decrease burnout [6].

Nevertheless, there are several barriers to PCPs performing manual procedures. In a study by Menahem et al., PCPs in Israel stated that lack of time, knowledge, and remuneration were the main barriers to performing minor surgical procedures [6]. Another study by Al-Ahaideb et al. found a lack of up-to-date skills and limited consultation time to be two main obstacles to performing minor musculoskeletal procedures in primary care [7]. These, among further studies, concluded the need for an improvement in the training programs of PCPs regarding procedural skills [6–9].

Procedural skills workshops for PCPs are not well studied in the current literature, and their effect is controversial. A small study conducted in New Zealand demonstrated that a procedural skills course positively impacted short-term confidence in performing these procedures, and this effect decreased unless there was ongoing clinical experience with the procedure [10]. On the contrary, a Canadian study conducted by MacKenzie et al. resulted in a lack of a significant increase in procedural skills performance after the participation of family medicine residents in a procedural skills workshop [11].

This study aimed to describe the procedural skills workshops program for PCPs, implemented in Maccabi Healthcare Services (MHS), the second largest HMO in Israel, and to investigate how many PCPs participated in each workshop since the program’s inception in 2017 and how many PCPs used the skills they have learned in these workshops.

## Methods

### Study design and setting

This observational study describes the implementation of a procedural skills workshops program in MHS between 2017 (when it was first launched) and 2021. We extracted all participants in courses that were held in these years. We also extracted from the electronic medical records (EMR) all procedures performed during these years by physicians in MHS. Each skill has its unique code actively filled by the physician after performing the procedure, based on which the compensation is made. We then connected each procedure with the physician who performed it and identified whether he was trained in MHS courses. The ethical committee of MHS (0071-22-MHS) approved the study, and all data was collected anonymously. Informed consent was waived and approved by the ethical committee of MHS (0071-22-MHS) due to the study design.

### Participants

All PCPs who enrolled in any procedural skills workshops from 2017 until 2021.

### Workshops

We examined five workshops, including dry needling, soft tissue, joint injections, BPPV (Epley maneuver), minor surgical procedures, and spirometry. All participants participated in at least one of the workshops on their own will, and participation was free of charge. After learning each procedure, PCPs who used them were paid according to MHS’ remuneration policy.

All workshops were in small groups and led by a trained tutor (who most often was also a PCP). The workshops were hands-on courses and required active participation by the PCPs attending the course. There was a constant evaluation process for each workshop using feedback questionnaires that were given to participants at the end of each workshop.

*Dry needling workshop* is a 25-hour workshop divided into three meetings. This course includes an introduction to myofascial pain syndrome and incorporates common trigger points’ dry needling techniques. The practical exercises were held in small groups of 6 or fewer participants.

*Soft tissue and joint injections workshop* – In this 12-hour course, PCPs studied aspiration and injection techniques in common anatomical structures, such as the knee, elbow, shoulder, plantar fascia, and carpal tunnel.

*BPPV workshop* – this is a 5-hour workshop that includes theoretical knowledge regarding the diagnosis and treatment of the patient who experiences dizziness as well as practicing maneuvers for the treatment of BPPV (such as the Epley maneuver). This workshop is led by an Ear Nose and Throat (ENT) specialist and a vestibular physiotherapist.

*Minor surgical procedures workshop* – this 6-hour workshop includes a theoretical and practical approach to treating ingrown toenails, abscesses incision and drainage, suturing and skin-glue usage, and burn care. Each class has 20 participants with three tutors.

*Spirometry workshop* – this is a 12-hour workshop, divided into two meetings. This workshop is led by an expert in lung diseases and a trained tutor.

### Variables

We report several variables for each year (during 2017–2021), including (a) the number of participants in each workshop, (b) the number of procedures performed for each skill learned (by physicians who underwent a course in MHS), (c) number of physicians who used the procedure they had learned, (d) the average number of procedures performed by each physician who took part in the workshop and (e) the average number of procedures performed by physicians who took part in the workshop and used the skill they had gained at least once.

### Statistical analysis

This is a descriptive study. We used the Mann-Kendall test to detect trends in procedures performed for each course during 2017–2021. The Mann-Kendall test is a non-parametric statistical test used to detect trends in data. Kendall’s Tau was calculated for each data series, along with the corresponding *p*-value. All analysis was made using Excel.

## Results

Between 2017 and 2021, 620 PCPs participated in workshops for dry needling, soft tissue and joint injections, BPPV, minor surgical procedures, and spirometry (Table 1).

**Table 1:** Participation and usage of different skills learned by primary care physicians during 2017–2021

Course	Year	Number of new participants	Overall, PCPs who learned the skill	Number of PCPs who used the learned skill	Average number of procedures performed by PCPs who used the skill at least once
**Dry needling**	2017	41	41	22	43
	2018	25	66	39	45.9
	2019	94	160	83	59.1
	2020	30	190	93	51.4
	2021	27	217	95	55.5
**Soft tissue and joint injections**	2017	34	34	16	16.5
	2018	76	110	18	15.9
	2019	16	126	26	15.7
	2020	18	144	24	17.3
	2021	26	170	23	18.8
**Vertigo maneuvers**	2017	52	52	27	5.8
	2018	98	150	63	6.7
	2019	49	199	83	8.9
	2020	68	267	92	8.8
	2021	18	285	109	7.5
**Minor surgical procedures**	2017	51	51	23	18.4
	2018	131	182	52	14.1
	2019	85	267	88	13.6
	2020	64	331	107	13
	2021	42	373	102	14.7
**Spirometry**	2017	19	19	1	1
	2018	20	39	7	8
	2019	50	89	18	9.3
	2020	23	112	24	6.8
	2021	0	112	16	7.4

*Dry needling - *217 PCPs participated in 12 workshops and performed 17,685 dry-needling procedures. Among the PCPs who participated in the workshops, 95 PCPs (43.8%) practiced the learned skill (i.e., used the learned skill at least once). The average annual number of procedures was 50.9 (for physicians who used the skill at least once).

*Soft tissue and joint injections workshop - 170 PCPs participated in 12 workshops and performed 1,807* procedures. Among the PCPs who participated in the workshops, 23 PCPs (13.5%) practiced the learned skill. The average annual number of procedures was 16.8 (for physicians who used the skill at least once).

*Vertigo maneuvers workshops* - There was a total of 285 participants in 16 BPPV workshops, and a sum of 2940 Epley maneuvers were performed. 109 (38.2%) PCPs practiced the learned procedure. The average annual number of procedures was 7.5 (for physicians who used the skill at least once).

*Minor surgical procedures workshops* - 373 PCPs participated in 23 workshops, and 5,245 procedures were performed. Among the workshop participants, 102 (27.3%) PCPs practiced the learned skill. The average annual number of procedures was 14.8 (for physicians who used the skill at least once).

*Spirometry* - Seven workshops included 112 participants, and 505 spirometry tests were performed. 16 (14.3%) PCPs practiced spirometry after participating in the workshop. The average annual number of procedures was 6.5 (for physicians who used the skill at least once)

**Fig. 1:**
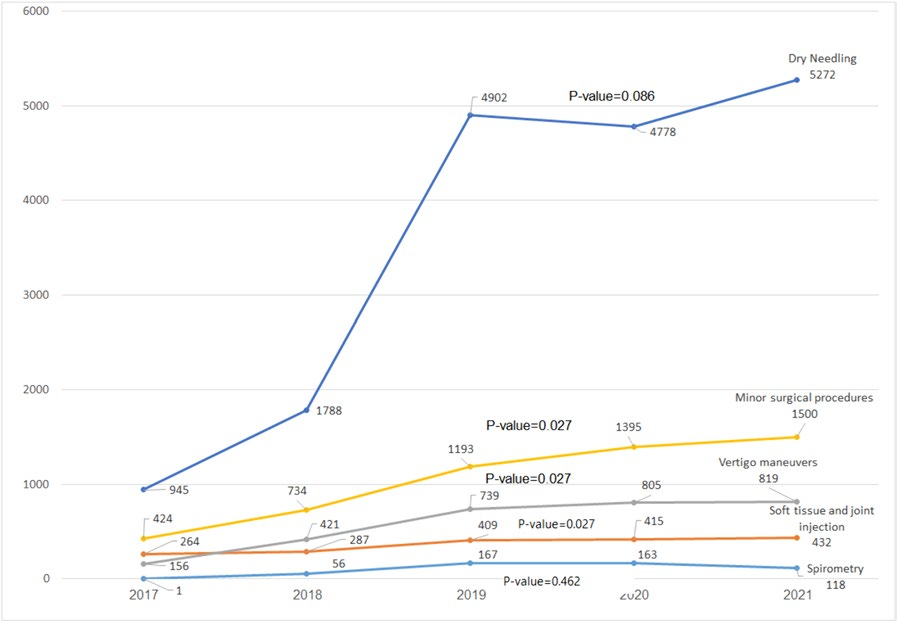
Number of procedures performed each year between 2017 and 2021 by physicians who learned it

The number of procedures per skill increased over the years (Fig. 1). The procedures for which the trend of increase was significant (*p*-value < 0.05) were minor surgical procedures, vertigo maneuvers, and soft tissue and joint injections. The skill most used by PCPs was dry needling, followed by minor surgical procedures.

**Fig. 2:**
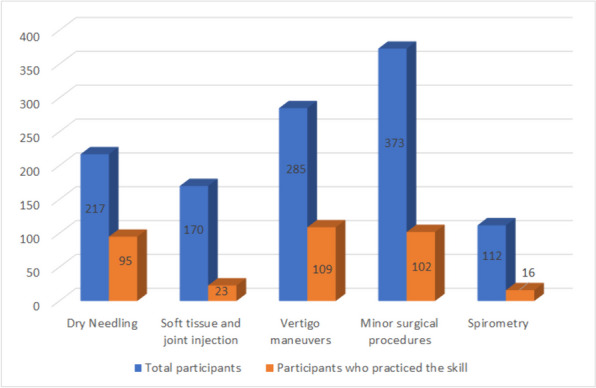
The number of physicians who learned the skill and used it at least once

For all procedures learned, less than half of all physicians used the skill (Fig. 2). The most used skill by PCPs who learned it was dry needling (used by 43.8%), treatment for vertigo (38.2%), and minor surgical procedures (27.3%). Spirometry and soft tissue and joint injections were used by less than 15% of the PCPs who learned the skill. Data regarding participants’ age, gender, and employment status was collected and shown in Table 2.

**Table 2:** Characteristics of primary care physicians participating in procedural skills workshops 2017–2021

	Dry needling	Soft tissue and joint injections	Vertigo maneuvers	Minor surgical procedures	Spirometry
**Age, mean (SD)**	45.5 (10.2)	45.1 (10.1)	46.5 (10.3)	43.7 (8.9)	49.4 (9.3)
**Gender**					
**Female n (%)**	130 (59.9)	88 (51.8)	147 (51.6)	110 (29.5)	35 (31.3)
**Male, n (%)**	85 (39.2)	78 (45.9)	97 (34)	84 (22.5)	37 (33)
**Missing data, n (%)**	2 (0.9)	4 (2.4)	41 (14.4)	179 (48)	40 (35.7)
**Employment status**					
**Salary**	138 (63.6)	91 (53.5)	134 (47)	113 (30.3)	32 (28.6)
**Fee for service**	73 (33.6)	55 (32.4)	72 (25.3)	53 (14.2)	26 (23.2)
**Missing data**	6 (2.8)	24 (14.1)	79 (27.7)	207 (55.5)	54 (48.2)

## Discussion

### Main findings

**Fig. 3:**
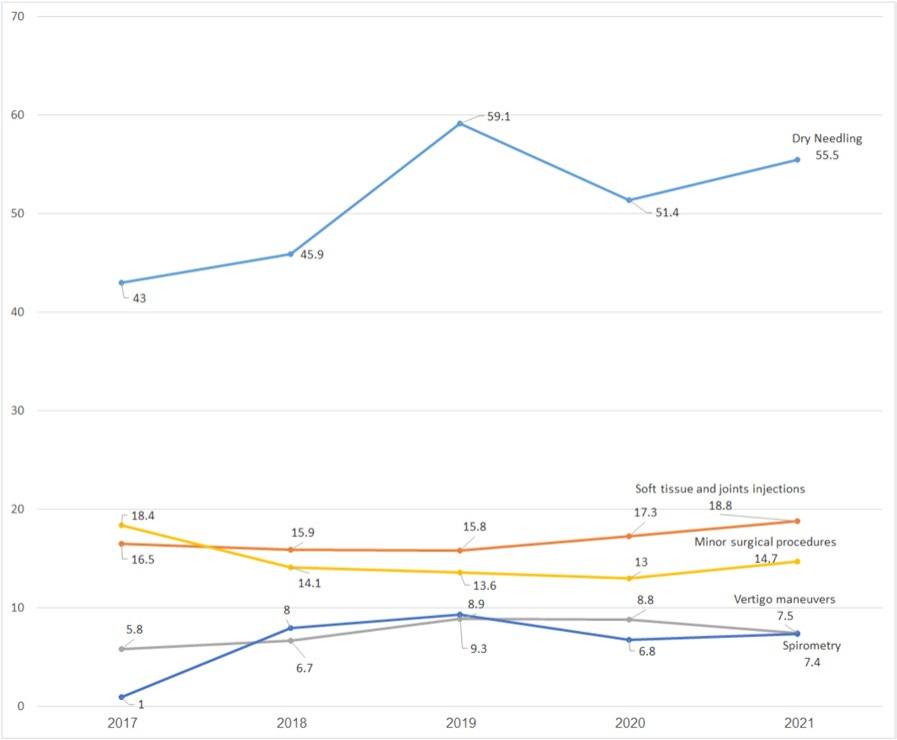
The average annual use of each skill per physician between 2017–2021

In this study, we present the implementation of a new program for procedural skills for PCPs in Israel. Five workshops were introduced, including dry needling, soft tissue and joint injections, BPPV treatment (Epley maneuver), minor surgical procedures, and spirometry. Between 2017 and 2021, participation increased, and an overall 620 PCPs participated in at least one course. Most procedures performed were dry needling (average annual number 3,537) and minor surgical procedures (average annual number 361). As seen in Fig. 3, the average annual use per physician was highest for dry needling (annual average use per physician who used the learned skill was 50.9), followed by soft tissue and joint injections (annual average use 16.8), minor surgical procedures (annual average use 14.8) and BPPV treatment (annual average use 7.5).

### Interpretation

Performing manual procedures by PCPs holds many advantages; from the patient’s perspective – The availability of a PCP is better than an ear, nose, and throat specialist (for example) for diagnosing and treating BPPV or a surgeon for diagnosing and treating an abscess. Furthermore, the patient’s continuity of care is optimal while being treated with the same medical provider – who will also perform a close follow-up and monitoring after the procedure [11, 12]. From the PCP’s perspective – the use of manual procedures may enhance potency and self-efficacy, reduce burnout, and save resources for the HMO [8].

In this study, some skills had a relatively high usage percentage (such as dry needling – 43.8%, Epley maneuver – 38.2%, and minor surgical procedures 27.3%), and skills with relatively low usage (soft-tissue injections – 13.5% and spirometry – 14.3%). There are a few factors that might contribute to this discrepancy.

For the dry needling and Epley maneuver – the procedures are relatively quick, do not require local anesthesia, and do not require the patient to acquire any further substances before the procedure is implemented. The effect of the procedures is immediate, with good responses and quick relief of pain or dizziness most often [13–15].

For soft tissue injections – in most cases, the patient needs to acquire an injectable corticosteroid and then make another appointment – which may encourage the PCP to refer the patient to an orthopedic surgeon for this procedure. In a cross-sectional study performed with primary care residents, 53.1% of residents performed soft-tissue injections during residency [7]. This high percentage compared with our study results (only 13.5% used this skill) might be related to the close mentoring and supervision while performing these procedures during residency, compared with the lack of these after the residency has ended.

Regarding the low spirometry usage rates (14.3%), this examination requires an office-based spirometer, which is not common in many PCP clinics and is expensive to purchase. In addition, during the COVID pandemic, the Israel Society of Pulmonology recommended not performing spirometry in the clinics, and therefore, PCPs also abstained from this procedure.

Less than 30% used minor surgical procedures. This is contrary to a higher usage rate shown in a Croatian study. In this study, a 40-hour surgical workshop increased abscess drainage and ingrown toenail removal rates, and 50% of the participants implemented the learned skills [16]. The increment in the usage percentage might be related to the difference in the workshop duration.

Regarding the PCPs’ procedural competency – there is a discrepancy between the different learned procedures, as seen in Table 1. For dry needling, minor surgical procedures, and soft tissue injections – PCPs perform the procedure at least once a month, which may be considered reasonable for maintaining manual capability. There is a relatively high usage rate in dry needling compared to other procedures; this may be because other procedures are also being performed by surgeons, ENT specialists, and physiotherapists. In contrast, Dry needling is performed mainly by PCPs and private physiotherapists, thus contributing to the high usage rates shown in this study.

Another noteworthy consideration is the need to keep up to date with the literature relating to these procedures. PCPs who perform procedural skills must consider this and devote time to reading and updating about the procedures they practice. This may be challenging to PCPs due to their need to keep up to date in many fields.

The obsolete ‘see one, do one, teach one’ approach might not work regarding procedural skills for PCPs [17] and should encourage further workshops to have more practicing time to increase usage and implementation. Refresher workshops are offered to PCPs who wish to preserve the learned skill. Hybrid workshops are offered to provide accessibility to PCPs nationwide.

### Strengths and limitations

The strengths of this study are the long time it covers (5 years) and the ability to follow the procedures performed by each participant for all relevant years. The limitations of this study are connected to its study design. Since this is a descriptive, observational study, we can only speculate about the reasons for using or not using the learned skill. We did not elaborate on who participated in each skill, which might have given more insight into understanding the low usage rate. We did not address the satisfaction rate of PCPs who used these skills and their patients’ satisfaction with them. Another important limitation of the study is that information regarding the procedures being performed was extracted according to the unique codes filled by the physicians in the EMR. Since reimbursement was related to the coding process, we assume that most physicians documented their procedures correctly, yet mis-coding might occur in some cases.

### Implications

Procedural skills workshops may expand PCPs’ therapeutic arsenal, thus empowering PCPs and providing more comprehensive care for patients. These workshops may be incorporated into continuous medical education (CME) programs, increasing the compliance rate of PCPs with the learned skills. Implementing these skills in PCPs’ routine work may improve continuity of care, one of the core elements of primary care. Lately, and perhaps related to the activity of the school, performing procedural skills was incorporated and made essential as part of the residency program in family medicine in Israel [18].

The incorporation of procedural skills in the residency program is a statement of the Israeli Medical Association that all PCPs should use these procedures. In real life, this is not the case. In order to start making this process, several changes and adaptations must be made at all levels of policymakers. First, the different teaching departments in each HMO should be able to teach residents these skills. For that, they should teach their senior physicians how to use procedural skills. However, this is not enough, as we demonstrated in this study. Learning the skill does not necessarily mean the physician will use it. So, different approaches should be used to ensure physicians use the learned skills, including refresher courses, along with lectures from practicing specialists in the field who discuss up-to-date evidence of the practice. Second, managers in all HMOs should provide courses to their PCPs (even those not teaching students or residents) so that using manual skills will become as broad as possible in the discipline of family medicine. Third, routing sessions should be offered at regular intervals to allow this process to continue and be assimilated into the organization. Fourth, it should be promoted to the public that when a patient needs an intervention, their family physician is the first to contact them.

In addition, this study shows the relatively high usage rates of certain skills and the paucity of other skills (such as spirometry and soft tissue injections). This may lead to a change in MHS’ policy – for example - regarding the need for an in-office spirometer as a threshold requirement for spirometry workshops and perhaps increase the availability of injectable corticosteroids in MHS clinics. Further evaluation regarding methods of improving participants’ compliance rates is needed.

## Conclusion

This study demonstrated the implementation of a procedural skills workshop program for PCPs in Israel. Many PCPs are interested in engaging procedural skills in their daily practice, as seen by the high number of participants in each course. Some procedural skills, such as dry needling, soft tissue injections, and the Epley maneuver, were more likely to be used by participants than other skills, such as Spirometry and soft tissue injections. This study may serve as a foundation for future studies regarding the difference between PCPs who practice the learned skills and those who don’t and the financial aspects of performing procedural skills by PCPs. Further studies are imperative to evaluate the impact of these workshops by addressing both physicians’ and patients’ satisfaction rates.

## Data Availability

The datasets generated and analyzed during the current study are not publicly available due to ethical restrictions. The datasets used and analyzed during the current study are available from the corresponding author upon reasonable request.

## Abbreviations

BPPV: Benign paroxysmal positional vertigo
CME: Continuous medical education
EMR: Electronic medical records
HMO: Healthcare maintenance organization
MHS: Maccabi Healthcare Services
PCPs: Primary care physicians
